# Relation of Plasma Catecholamine Concentrations and Myocardial Mitochondrial Respiratory Activity in Anesthetized and Mechanically Ventilated, Cardiovascular Healthy Swine

**DOI:** 10.3390/ijms242417293

**Published:** 2023-12-09

**Authors:** Nadja Abele, Franziska Münz, Fabian Zink, Michael Gröger, Andrea Hoffmann, Eva-Maria Wolfschmitt, Melanie Hogg, Enrico Calzia, Christiane Waller, Peter Radermacher, Tamara Merz

**Affiliations:** 1 Institute for Anesthesiological Pathophysiology and Process Engineering, Ulm University Medical Center, 89069 Ulm, Germany; nadja.abele@uni-ulm.de (N.A.); fabian.zink@uni-ulm.de (F.Z.); michael.groeger@uni-ulm.de (M.G.); andrea.hoffmann@uni-ulm.de (A.H.); eva-maria.wolfschmitt@uni-ulm.de (E.-M.W.); melanie.hogg@uni-ulm.de (M.H.); enrico.calzia@uni-ulm.de (E.C.); 2 Clinic for Anesthesiology and Intensive Care, Ulm University Medical Center, 89069 Ulm, Germany; 3 Clinic for Psychosomatic Medicine and Psychotherapy, Paracelsus Medical Private University, 90402 Nuremberg, Germany; christiane.waller@klinikum-nuernberg.de

**Keywords:** plasma catecholamines, cardiac tissue, mitochondrial respiration, β_2_-adrenoreceptor, reactive oxygen species

## Abstract

Chronic heart failure is associated with reduced myocardial β-adrenergic receptor expression and mitochondrial function. Since these data coincide with increased plasma catecholamine levels, we investigated the relation between myocardial β-receptor expression and mitochondrial respiratory activity under conditions of physiological catecholamine concentrations. This post hoc analysis used material of a prospective randomized, controlled study on 12 sexually mature (age 20–24 weeks) Early Life Stress or control pigs (weaning at day 21 and 28–35 after birth, respectively) of either sex. Measurements in anesthetized, mechanically ventilated, and instrumented animals comprised serum catecholamine (liquid-chromatography/tandem-mass-spectrometry) and 8-isoprostane levels, whole blood superoxide anion concentrations (electron spin resonance), oxidative DNA strand breaks (tail moment in the “comet assay”), post mortem cardiac tissue mitochondrial respiration, and immunohistochemistry (β_2_-adrenoreceptor, mitochondrial respiration complex, and nitrotyrosine expression). Catecholamine concentrations were inversely related to myocardial mitochondrial respiratory activity and β_2_-adrenoceptor expression, whereas there was no relation to mitochondrial respiratory complex expression. Except for a significant, direct, non-linear relation between DNA damage and noradrenaline levels, catecholamine concentrations were unrelated to markers of oxidative stress. The present study suggests that physiological variations of the plasma catecholamine concentrations, e.g., due to physical and/or psychological stress, may affect cardiac β_2_-adrenoceptor expression and mitochondrial respiration.

## 1. Introduction

Activation of the sympatho-adrenergic system is crucial for the short-term adaptation to stress, e.g., critical illness. Accordingly, exogenous noradrenaline is the drug of first choice for the management of circulatory shock. However, in patients [1,2] as well as in large-animal models, like swine [3,4,5,6,7], the sustained over-activation of the sympathetic system, and consecutive elevation of plasma adrenaline and noradrenaline levels associated with chronic heart failure [8], is detrimental by ultimately potentially triggering cardiotoxicity [9]. In addition to the potential development of myocardial O_2_ supply/demand imbalance, catecholamine-related cardiotoxicity has been associated with mitochondrial dysfunction and/or damage resulting from (i) β-adrenoceptor activation-mediated Ca^++^-overload and (ii) oxidative stress due to abnormal activation of the non-phagocytic NAD(P)H oxidase in response to norepinephrine [10], as well as (iii) transformation of catecholamines into “aminochromes” undergoing redox cycling in mitochondria to excessively generate reactive oxygen species (ROS) [8,9]. In fact, even in patients with heart disease with no or only mild heart failure, Lemieux et al. demonstrated defective Complex I-linked respiration using high-resolution respirometry [11]. The authors also showed that progression to heart failure was associated with aggravated impairment of oxidative phosphorylation and electron transfer capacity, which additionally coincided with a decline in mitochondrial density [11]. These findings agree well with data from canine chronic heart failure induced by repetitive coronary micro-embolization: plasma noradrenaline was 2–3-fold higher than in control animals, and the “mitochondrial injury index” showed a significant direct relation to the noradrenaline levels [12].

The chronic heart failure-related sustained elevation of endogenous noradrenaline levels also led to β_1_-adrenoceptor down-regulation and desensitization [1,2,13,14,15], while β_2_-adrenoceptor expression remained unaltered [15,16]. This, in turn, was accompanied by a decreased response to β-receptor agonists including that of endogenous catecholamines [17]. In fact, in patients with cardiomyopathy, a significant direct relation was demonstrated between the left ventricular β-receptor concentration as assessed using positron emission tomography and the contractile response to intracoronary dobutamine application [2]. It is of note in this context that the crucial role of catecholamine-induced β-adrenoceptor activation for both mitochondrial dysfunction and excess ROS formation was further demonstrated by various experimental models using exogenous administration of adrenaline, noradrenaline and synthetic derivatives (e.g., isoproterenol), and the respective receptor antagonists in rats and dogs [18,19,20,21,22]. Finally, Perez recently highlighted the crucial role of adrenergic receptors as potential therapeutic targets to increase cardiac mitochondrial respiratory capacity and lower ROS production [15].

In contrast to the fairly consistent response pattern of plasma catecholamine concentrations and myocardial β-receptor density during chronic heart failure, acute challenges produced more variable results: in rats, arterial hypotension with mean arterial blood pressures ≈45–50 mmHg resulting from injection of live *E. coli* [23] or hemorrhage [24] caused a several-fold increase in plasma adrenaline and noradrenaline levels. In contrast, myocardial β-receptor density was either unchanged [23] or markedly reduced [24]. Nevertheless, regardless of whether dogs or pigs were studied immediately [25] or 2–3 weeks [26] after coronary micro-embolization, heart failure was invariably associated with impaired oxidative phosphorylation in left ventricular tissue specimens.

As mentioned above, all data on altered myocardial β-receptor density and/or mitochondrial respiration were reported under conditions of supra-normal plasma catecholamine concentrations. To our knowledge, no data are available regarding whether plasma catecholamine levels within the physiological range, e.g., resulting from psychological and/or physical stress, may also impact myocardial β-receptor density and/or mitochondrial respiratory activity. Therefore, in the present study, we investigated the relation between plasma adrenaline and noradrenaline levels, blood markers of ROS production and oxidative stress, as well as cardiac tissue β-receptor expression, mitochondrial respiratory complex expression, and mitochondrial respiratory activity. The data presented are a post hoc analysis of material available from a study published previously [27].

## 2. Results

Table 1 summarizes the data of the blood catecholamine, isoprostane, and superoxide anion concentrations as well as the DNA single-strand breaks presented as the “tail moment” in the comet assay. All values were in the lower physiological range reported by other authors in porcine blood samples for catecholamines [28] and isoprostanes [29] as well in as the “tail moment” of the comet assay [30,31], and in healthy volunteers for superoxide anion blood concentrations as assessed using electron spin resonance [32,33].

**Figure 1:**
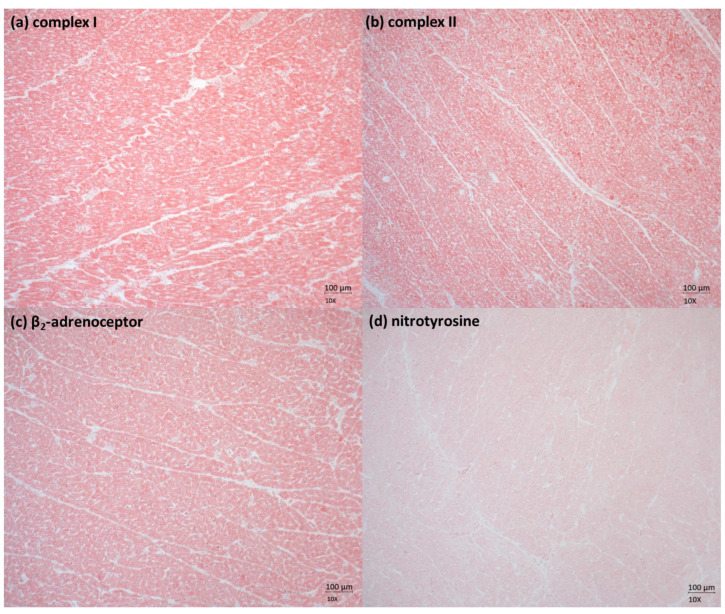
Immunohistochemistry examples of the cardiac tissue protein expression of the (**a**) mitochondrial NADH dehydrogenase (ubiquinone oxidoreductase, complex I); (**b**) succinate dehydrogenase (complex II); (**c**) β_2_-adrenergic receptor, and (**d**) nitrotyrosine formation. Note that no nitrotyrosine staining could be detected, indicating that cardiac tissue oxidative and nitrosative stress was negligible if present at all. All pictures are displayed with a magnification of 10×.

Figure 1 and Table 2 show representative pictures (Figure 1) as well as the overall quantitative analysis (Table 2) of the tissue protein expression of subunits of the mitochondrial NADH:ubiquinone oxidoreductase (Complex I; Figure 1a), succinate dehydrogenase (Complex II; Figure 1b), and the β_2_-adrenergic receptor (Figure 1c) as assessed using immunohistochemistry. Sulfide quinone oxidoreductase, Complex III, and Complex IV expression were not quantified because visual examination of the samples revealed no obvious differences in the intensity of the staining between the individual animals. Nitrotyrosine formation was not quantified, since no nitrotyrosine formation was detected in any of the heart tissue specimens. Hence, data for these markers are not shown. In addition, Table 2 also shows the overall data of the maximum respiratory capacity in the coupled state (OxPhos) and the maximum electron transfer capacity in the uncoupled state (ETC) of the myocardial mitochondrial respiratory activity (presented as JO_2_). The latter were within the normal range for healthy swine as reported by others [34,35].

**Figure 2:**
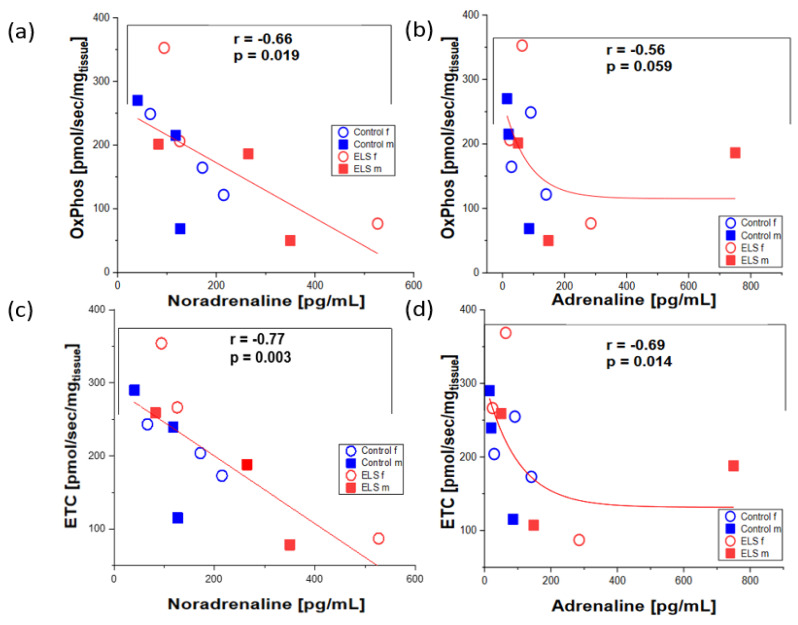
(**a**) Myocardial tissue maximum respiratory capacity in the coupled state (OxPhos) plotted as a function of the noradrenaline concentration; (**b**) myocardial tissue OxPhos plotted as a function of the adrenaline concentration; (**c**) myocardial tissue maximum electron transfer capacity in the uncoupled state (ETC) plotted as a function of the noradrenaline concentration, and (**d**) myocardial tissue ETC plotted as a function of the adrenaline concentration. Males are presented by closed squares, females by open circles, presence or absence of early life stress (ELS) is depicted by red and blue symbols, respectively. While OxPhos and ETC showed a significant, inverse linear correlation with the noradrenaline levels (r = −0.68, *p* = 0.015, and r = −0.76, *p* = 0.004, respectively), a significant, inverse non-linear correlation was present for ETC as a function of the adrenaline concentrations (r = −0.69, *p* = 0.013). The inverse non-linear relation of OxPhos plotted as a function of the adrenaline levels narrowly missed statistical significance (r = −0.57, *p* = 0.051).

Figure 2 shows the myocardial tissue OxPhos and ETC plotted as a function of the noradrenaline and adrenaline concentrations. While OxPhos and ETC showed a significant, inverse linear correlation with the noradrenaline levels (r = −0.68, *p* = 0.015, and r = −0.76, *p* = 0.004, respectively); a significant, inverse non-linear correlation was present for ETC as a function of the adrenaline concentrations (r = −0.69, *p* = 0.013). The inverse non-linear relation of OxPhos plotted as a function of the adrenaline levels narrowly missed statistical significance (r = −0.57, *p* = 0.051).

**Figure 3:**
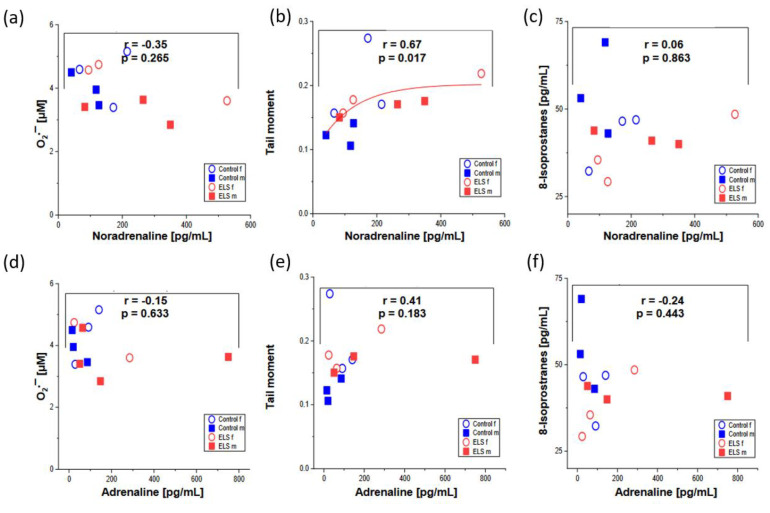
(**a**) Whole blood superoxide anion (O_2_^•−^) concentrations; (**b**) whole blood tail moment in the comet assay, and (**c**) plasma isoprostane levels plotted as function of the noradrenaline concentration, and (**d**–**f**) the same parameters of oxidative stress plotted as a function of the adrenaline concentration. Males are presented by closed squares, females by open circles, presence or absence of ELS is depicted by red and blue symbols, respectively. Except for a significant, direct, non-linear correlation between the tail moment and the noradrenaline levels (r = 0.67, *p* = 0.017), no significant relation was detected.

Figure 3 shows the markers of oxidative stress (whole blood superoxide anion O_2_^•−^ concentrations, whole blood tail moment in the comet assay, and plasma isoprostane levels) plotted as function of the noradrenaline and adrenaline concentrations. Except for a significant, direct, non-linear correlation between the tail moment and the noradrenaline levels (r = 0.67, *p* = 0.017), no significant relation could be detected.

**Figure 4:**
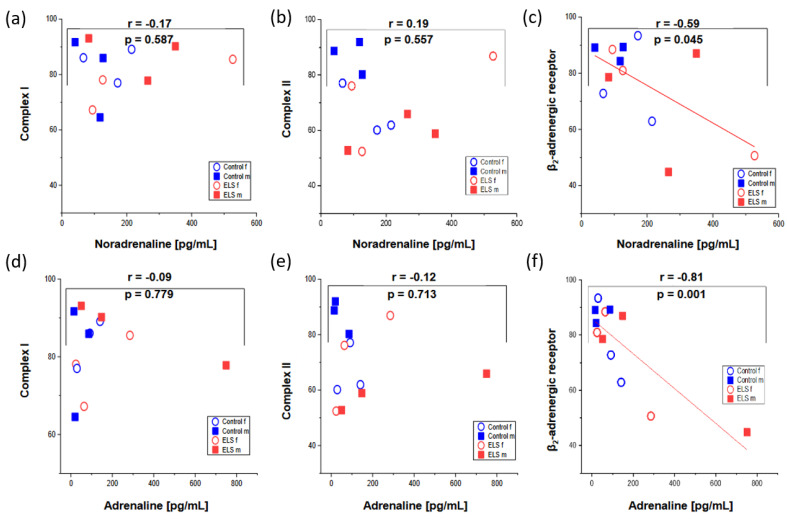
Results of the immunohistochemistry quantification of the myocardial tissue expression of the (**a**) mitochondrial respiratory complex I, (**b**) mitochondrial respiratory complex II, and (**c**) β_2_-adrenergic receptor plotted as a function of the noradrenaline; and (**d**–**f**) the same parameters plotted as a function of the adrenaline concentrations. Males are presented by closed squares, females by open circles, presence or absence of ELS is depicted by red and blue symbols, respectively. While there was no significant relation between the expression of the mitochondrial respiratory complexes and the catecholamine levels, the expression of the β_2_-adrenergic receptor showed a significant, inverse, linear correlation with both the noradrenaline (r = −0.59, *p* = 0.045) and adrenaline (r = −0.81, *p* = 0.001) concentrations.

Figure 4 shows the results of the immunohistochemistry quantification of the myocardial tissue expression of the subunits of the mitochondrial respiratory complexes I and II as well as the β_2_-adrenergic receptor plotted as a function of the noradrenaline and adrenaline concentrations. While there was no significant relation between the expression of the mitochondrial respiratory complexes and the catecholamine levels, the expression of the β_2_-adrenergic receptor showed a significant, inverse, linear correlation with both the noradrenaline (r = −0.59, *p* = 0.045) and adrenaline (r = −0.81, *p* = 0.001) concentrations.

## 3. Discussion

It is well established that chronic heart failure is associated with a sustained increase of plasma catecholamine levels, which, in turn, coincides with reduced myocardial β_2_-adrenergic receptor density and impaired cardiac tissue mitochondrial function. Increased catecholamine concentrations resulting from acute challenges, like circulatory shock, and/or exogenous catecholamine administration resulted in a similar response pattern. Since all data on altered myocardial β-receptor density and/or mitochondrial respiration were reported under conditions of supra-normal plasma catecholamine concentrations, we aimed to answer the question whether plasma catecholamine levels under physiological, i.e., stress-free conditions and in the absence of exogenous catecholamine administration may also impact myocardial β-receptor expression and/or mitochondrial respiratory activity. The main findings of this study were that even physiological catecholamine concentrations showed significant inverse relationships with both (i) myocardial mitochondrial respiratory activity and (ii) tissue expression of the β_2_-adrenergic receptor, whereas (iii) no relation was found between catecholamine concentrations and the expression of mitochondrial respiratory complexes.

In our animals, median plasma noradrenaline and adrenaline concentrations were 127 and 75 pg/mL. Except for a single adrenaline level, all individual values were below the upper threshold of the normal range reported for pigs, i.e., 800 and 300 pg/mL, respectively [28]. Moreover, these concentrations well agree with those reported for both anesthetized and mechanically ventilated pigs under baseline conditions [36] as well as awake, “non-stress susceptible” individual swine prior to transport stress [37]. Similar catecholamine levels were also reported in adult swine prior to induction of a “hypertension+hyperlipidemia-induced heart failure with preserved ejection fraction”, and the range of variation in that study resembled that in our experiment [38]. Finally, these noradrenaline and adrenaline concentrations are 4–7-fold and 6-fold lower than those in swine with rapid pacing-inducing congestive heart failure [3,4,5,6,7,39]. Together with the cortisol concentrations of 65 ± 24 ng/mL as reported in the original publication [27], which were also within the normal range of morning values [28,40], we can exclude any stress response induced by the experimental procedures. Hence, albeit recorded in anesthetized, mechanically ventilated animals that had undergone some surgical instrumentation rather than in awake animals, our data represent strictly physiological, un-stressed conditions.

We found significant inverse relationships between myocardial tissue mitochondrial respiratory capacity and plasma catecholamine concentrations. It is well-established that impairment of myocardial mitochondrial respiration occurs during chronic heart failure as well as acute challenges resulting from circulatory shock and/or exogenous administration of catecholamines, i.e., under conditions of supra-normal plasma catecholamine concentrations. To our knowledge, our findings are the first to report that even catecholamine levels within the normal physiological range may directly affect cardiac mitochondrial respiratory capacity. In other words, our data suggest that even acute variations in endogenous catecholamine release under physiological conditions and without exogenous catecholamine administration, e.g., exposure to psychological stress [41], may interfere with cardiac mitochondrial respiration. Psychological stress, on the one hand, is associated with increased plasma catecholamine concentrations [42,43,44] and, on the other hand, affects immune cell mitochondrial respiration [45,46,47,48,49]. However, so far, a direct interaction of endogenous variations in catecholamine concentrations within the physiological range and tissue mitochondrial respiration has only been documented for skeletal muscle in swine undergoing cold exposure [50].

Although there were significant inverse correlations between mitochondrial respiratory capacity and plasma catecholamine concentrations, no relation was detectable between the expression of the mitochondrial complexes I (NADH:ubiquinone oxidoreductase) and II (succinate dehydrogenase), suggesting that any effect on myocardial tissue OxPhos and/or ETC was not due to variable mitochondrial density and/or expression of respiratory proteins. This finding is in good agreement with previous data in porcine acute [35] or chronic myocardial dysfunction [51]: 24 h of fecal peritonitis was associated with significant impairment of complex II and IV activity, while citrate synthase activity, a marker of mitochondrial content, remained unaffected [35]. Moreover, in young adult miniature swine with heart failure induced by 20 weeks of aortic banding, complex I dysfunction coincided with unchanged expression of complex I and IV [51]. Finally, in swine with streptozotocin-induced diabetes type I, the impaired left ventricular ETC and increased myocardial superoxide anion production were associated with only very minor reductions in mitochondrial density protein or content [34].

In contrast to the lacking relation between the expression of mitochondrial respiratory proteins and plasma catecholamine concentrations, we found significant inverse linear correlations between the cardiac tissue expression of the β_2_-adrenergic receptor and the plasma catecholamine concentrations. It is well-established that chronic heart failure and the consecutive sustained increase in plasma catecholamine concentrations is associated with down-regulation of the cardiac β_1_-receptors [13,14,15], while β_2_-receptor expression remains unaffected [15,16]. This may ultimately lead to decreased responses to β-adrenoceptor agonists [2,17]. In animal models, equivocal data are available on the impact of increased plasma adrenaline and/or noradrenaline concentrations on the myocardial β-adrenoceptor expression, inasmuch as unchanged [23,52], reduced [18,53], or even increased [19] (β_1_) receptor expression were reported. Results depended on whether challenges were acute [19,23] or maintained over days and weeks [18,52,53] and/or whether exogenous catecholamine administration [18,19] had been studied. Acute increases in plasma catecholamine levels are normally associated with increased mitochondrial respiratory enzyme activity, and, hence, OxPhos and/or ETC [54], e.g., during physical exercise [55]. Our data suggest that even variations in the plasma catecholamine concentrations within the normal physiological range may affect the cardiac tissue β_2_-adrenergic receptor expression and, thereby possibly the response to β-adrenoceptor agonists.

In chronic heart failure, the sustained elevation of plasma adrenaline and noradrenaline levels resulting from over-activation of the sympathetic system was associated with aggravated oxidative stress due to abnormal activation of the non-phagocytic NAD(P)H oxidase in response to noradrenaline [10] and transformation of catecholamines into “aminochromes” undergoing redox cycling in mitochondria to excessively generate ROS [8,15]. Furthermore, the 2–3-fold increases in plasma noradrenaline levels in canine chronic heart failure induced by coronary micro-embolization showed a significant relation to the “mitochondrial injury index” [12]. Finally, in various animal models, exogenous catecholamine administration was associated with induction of oxidative stress [19,20,56], in particular as a result of noradrenaline-induced excess superoxide anion formation [57]. In our experiment under conditions of catecholamine levels within the normal physiological range, we did not find any relation between blood superoxide anion, plasma isoprostane concentrations, or tissue nitrotyrosine formation and plasma noradrenaline or adrenaline levels. Whole blood superoxide anion blood concentrations and plasma isoprostane levels were comparable to those reported for healthy volunteers [32,33], as well as both swine [29] and control patients [58] when using the same methodology as in our present study. Moreover, we did not find any tissue nitrotyrosine formation. Hence, if present at all, there was only a minor degree of oxidative stress on both the systemic and the cardiac tissue level. Nevertheless, the “tail moment” in the comet assay, i.e., whole blood oxidative DNA strand breaks, showed a significant, non-linear direct relationship with plasma noradrenaline levels, albeit also well within the normal range reported for healthy volunteers [30,31]. This result agrees well with our previous study in healthy volunteers: the psychological stress induced by the Trier social stress test for groups (TSST-G) was not only associated with a moderate rise in salivary α-amylase activity, which was assessed as a surrogate for noradrenaline plasma concentrations, but also with a mild increase in the tail moment indicating mild oxidative DNA damage [41]. Other authors reported increased left ventricular tissue superoxide anion formation in adult minipigs with streptozotocin-induced diabetes mellitus; however, animals were studied after five months [34]. Nevertheless, since we did not find any increase in markers of enhanced oxidative stress in the cardiac specimens, it remains speculative whether increased ROS formation contributed to our finding of an inverse relation between cardiac mitochondrial respiration and catecholamine concentrations.

### Limitations of the Study

Our study is certainly limited by the fact that for this pilot experiment, we were only able to obtain a permission from the Animal Care Committee of the Universität Ulm and the Federal Authorities for Animal Research (Regierungspräsidium Tübingen) for six control and six ELS animals. Therefore, a power calculation was unavailable, and, consequently, more significant differences may have been missed due to the small number of pigs in each group. In addition, studying pigs per se may limit the transferability of our results; however, several porcine studies available in the literature have demonstrated the usefulness of swine as a surrogate for human physiology with respect to cardiac function and markers of oxidative as well as stress hormone plasma concentrations [3,25,27,28,29,39,40]. Clearly, studying anesthetized and mechanically ventilated animals after minor surgical instrumentation rather than awake pigs may have influenced the results. However, this approach was explicitly chosen in order to (i) control as much as possible parameters of hemodynamics, gas exchange, acid-base status, and metabolism, and, in accordance with the “3R” principle of animal experimentation, (ii) simultaneously avoid as much as possible any stressful conditions. In fact, as mentioned above, the stress hormone plasma concentrations prove that our data represent a strictly physiological, un-stressed situation. Finally, it could be argued that exogenous administration of catecholamines might have allowed for more definitive assessment of possible cause/effect relationships. Again, we explicitly did not choose this approach in order to study physiological conditions rather than a situation with possible catecholamine-induced changes in particular in hemodynamics (e.g., tachycardia, and/or arterial hypertension) and metabolism (e.g., increased oxygen demand, hyperglycemia, and/or -lactatemia) that would have per se influenced cardiac energy metabolism.

## 4. Materials and Methods

The data presented are a post hoc analysis of material available from a previously published study [27] in order to comply with the “3R” principle of animal experimentation requesting the minimization of animal numbers used. The original experiments had been performed after obtaining the approval by the University of Ulm Animal Care Committee and the Federal Authorities for Animal Research (Regierungspräsidium Tübingen; Reg.-Nr. 1559, approval 29 October 2021) and in compliance with the National Institute of Health Guidelines on the Use of Laboratory Animals and the European Union “Directive 2010/63/EU on the protection of animals used for scientific purposes”. The data presented are from twelve young (median (interquartile range) age 23 (22; 24) weeks, bodyweight 82 (67; 93) kg), sexually mature German Large White pigs with equal sex distribution (*n* = 3 males/females each per group). Animals of the “control” group had been weaned on day 28–35 after birth, which corresponds to the weaning period regularly used for swine husbandry. In contrast, swine with “early life stress (ELS)” had already been weaned on day 21 after birth. This time point had been chosen, because (i) it represents the earliest time point for swine weaning described in the Federal German regulations on farm animal husbandry (“Tierschutz-Nutztierhaltungsverordnung—TierSchNutztV, 22 August 2006; last amendment 29 January 2021), and (ii) was evaluated as “*…not to cause any violation of animal protection…*” according to the chapter no. 90 entitled “*Influence of weaning age on piglet behaviour*” (“*Einfluss des Absetzalters auf das Verhalten von Ferkeln nach dem Absetzen*”) of the report on “*Environmentally compatible and site-specific agriculture*” (“*Umweltverträgliche und Standortgerechte Landwirtschaft*”) of the Friedrich-Wilhelms-University, Bonn, Germany. Moreover, (iii) we aimed to avoid any pathological clinical symptoms associated with earlier (at day 16–18) weaning of piglets in other ELS models, e.g., diarrhea, weight loss, and/or intestinal mucosal barrier dysfunction [59,60,61,62]. In order to minimize inter-individual differences with respect to age and development as far as possible, every 2 pairs of control and ELS animals had been taken from the same litter. Since neither the analysis of heart tissue mitochondrial respiratory activity or protein expression nor plasma catecholamine levels showed any effect of sex or of presence/absence of ELS, data of all 12 animals were pooled.

### 4.1. Anesthesia and Surgery

Anesthesia and surgery have been described in detail in the previously published study [27]. Briefly, animals had their last meal at the evening before the experiment and free access to water during the 12 h preceding the experiment. In the morning of the experimental day, pigs received an intramuscular pre-medication (2 mg/kg of azaperone plus 0.5–1 mg/kg of midazolam), followed by placement of a peripheral venous catheter in an ear vein. Subsequently, general anesthesia was induced using propofol (1.5–2 mg/kg) and ketamine (1 mg/kg), followed by endotracheal intubation, and fentanyl (20 µg/kg). Muscle paralysis was achieved using pancuronium (0.1 mg/kg). Animals were mechanically ventilated using the following ventilator settings: tidal volume 8 mL/kg, respiratory rate 8–12 breaths/minute adapted to achieve an arterial PCO_2_ (PaCO_2_) = 35–40 mmHg, inspiratory/expiratory ratio (I/E) of 1:1.5, fraction of inspiratory oxygen (FiO_2_) of 0.3, positive end-expiratory pressure 10 cmH_2_O to prevent atelectasis formation [27]. Anesthesia was maintained by continuous intravenous infusion of propofol (10 mg/kg/h). A balanced electrolyte solution (10 mL/kg/h, Jonosteril 1/1^®^, Fresenius Kabi, Bad Homburg, Germany) was infused as maintenance fluid. Via surgical cut down, a 9F-metal-sheathed catheter (Arrow^®^ International Inc. (Teleflex), Morrisville, NC, USA) was placed in the left iliac artery for continuous blood pressure monitoring and blood sampling. After completion of the surgical instrumentation, ventilator settings were modified to an I/E ratio = 1:2, FiO_2_ = 0.21, and zero end-expiratory pressure (0 cmH_2_O) to mimic physiological conditions as much as possible.

### 4.2. Experimental Protocol

All experiments had followed a strict timeline, thus allowing to avoid any effect of circadian rhythm, i.e., intramuscular pre-medication had always been performed at 06:00 h, induction of general anesthesia at 07:00 h, and, subsequently, surgical instrumentation for approximately 45 min. As described in the previously published study [27], arterial blood sampling was performed one hour after surgical instrumentation, immediately followed by euthanization with KCl after anesthesia had been further deepened for immediate post mortem organ sampling. Overall, individual experiment duration did not vary by >15 min.

### 4.3. Measurements and Calculations

In addition to recording of blood temperature, heart rate, and mean arterial pressure, arterial blood samples were taken for the measurement of PaO_2_ and PaCO_2_, acid–base status, and metabolic parameters (lactate, glucose). Catecholamine levels (adrenaline, noradrenaline) were determined after centrifugation of whole blood samples in Li^+^-heparine-coated, stabilizer-primed (20 µL/mL blood containing 0.2 M reduced glutathione and 0.2 M ethylenglycol-bis(aminoethylether)-N,N,N′,N′-tetra-acetic acid (EGTA), both Carl-Roth, Karlsruhe, Germany) tubes using liquid-chromatography/tandem-mass-spectrometry (LC-MS/MS) (external analysis by Dr. Eberhard & Partner, Dortmund, Germany). In one animal, adrenaline concentrations were below the detection limit of 15 pg/mL. For this animal, this lower threshold value was used for analysis.

Heart tissue mitochondrial respiration was measured by high-resolution respirometry using the Oxygraph-2K^®^ (Oroboros Instruments, Innsbruck, Austria). This device allows for simultaneous recording of the O_2_ concentration in two parallel chambers calibrated for 2 mL of respiration medium MiR05 [63,64]. This medium is composed of 110 mM D-Sucrose (Sigma Aldrich, St. Louis, MO, USA), 60 mM K-Lactobionate (Sigma Aldrich, St. Louis, MO, USA), 0.5 mM ethylene glycol tetra acetic acid (Sigma Aldrich, St. Louis, MO, USA), 1 g/L bovine serum albumin free from essentially fatty acids (Sigma Aldrich, St. Louis, MO, USA), 3 mM MgCl_2_ (Scharlau, Hamburg, Germany), 20 mM taurine (Sigma Aldrich, St. Louis, MO, USA), 10 mM KH_2_PO_4_ (Merck, Darmstadt, Germany), and 20 mM HEPES (Sigma Aldrich, St. Louis, MO, USA), adjusted to pH = 7.1 with KOH and equilibrated with 21% O_2_ at 37 °C. Heart tissue homogenates containing 0.75 mg tissue/mL of respiration medium were filled into both chambers and continuously stirred at 750 rpm. Closing the chambers by gently pushing down the stoppers started the continuous recording of mitochondrial respiration, which was quantified in terms of O_2_ flux (JO_2_) based on the rate of change of the O_2_-concentration in the chambers normalized for tissue weight. Once the chambers were sealed, specific analysis of mitochondrial respiratory function was achieved by sequential injections of mitochondrial substrates and inhibitors into the respiration medium. Recording of measurements started after achieving a stable JO_2_-signal. The maximum respiratory capacity in the coupled state (OxPhos) was determined after the addition of 2 mM malate, 10 mM glutamate, 5 mM ADP, 5 µM cytochrome c, 10 mM pyruvate, 1 mM octanoyl-carnitine, and 10 mM succinate, and the maximum respiratory capacity in the uncoupled state (ETC) was measured after the repetitive titration of 1 µM FCCP. The data shown are normalized for tissue wet weight.

Whole blood superoxide anion (O_2_^•−^) concentrations were determined immediately after sampling as described previously [27,63]. For this purpose, 25 µL of whole blood was mixed with an aliquot of 25 µL freshly thawed CMH spin probe solution. The CMH solution contained 400 µM CMH spin probe (1-Hydroxy-3-methoxycarbonyl-2,2,5,5-tetramethylpyrrolidine), 25 µM deferoxamine, and 5 µM diethyldithiocarbamate to chelate transition metal ions in Krebs-HEPES-Buffer (KHB) (Noxygen, Elzach, Germany). After mixing whole blood with CMH, the solution was transferred to a 50 µL glass capillary, sealed, and measured with an EMXnano electron spin resonance (ESR) spectrometer (Bruker, Billerica, MA, USA) after 5 min incubation at 37 °C (Bio-III, Noxygen, Elzach, Germany). For each measurement, 3 scans of the following settings were averaged: 3440 G center field, 60 G sweep width, 72.70 ms conversion time, 9.66 GHz microwave frequency, 0.3162 mW microwave power, and 2 G modulation amplitude. As a blank sample, KHB added to the respective amount of CMH was measured and subtracted from the sample value.

As a marker of oxidative stress, whole blood DNA single strand-breaks were quantified as “tail moment” using single cell gel electrophoresis (alkaline version of the “comet assay”) adapted for swine blood as described previously [41,65]. For this purpose, 5 µL whole blood was mixed with 120 mL LMP-Agarose (37 °C) and applied on a slide. Slides were stored in lysis-buffer for 2 days at 4 °C. Briefly after lysis, the cells were denaturated with alkali (electrophoresis buffer pH 13) for 40 min, followed by electrophoresis for 40 min at 25 V and 300 mA. Slides were stained with 50 µL ethidium bromide and evaluated by image analysis (Comet Assay II, Perceptive Instruments, Haverhill, UK). Results are shown as mean tail moment (percentage of DNA in the tail × tail length) according to the image analysis software (Comet Assay II V1.02).

Immunohistochemistry was used to quantify myocardial expression of the β_2_-adrenoreceptor, subunits of the mitochondrial respiration complexes I–IV, and nitrotyrosin as a marker of tissue oxidative and nitrosative stress. Immunohistochemistry was chosen, because (i) it is well established in the literature that densitometric analysis of colorimetric immunohistochemical staining is as acceptable a method as Western blotting for protein measurement [66], (ii) we had previously established the immunohistochemistry protocols for porcine cardiac tissue specimens [67,68], (iii) we had obtained highly significant correlations between the densitometric values and those obtained by Western blotting [69], and (iv) in contrast to Western blotting, the immunohistochemistry evaluation of the tissue allows identification of the physical topography and protein expression in different cell types within the tissue specimen. Immunohistochemistry of heart specimens was performed as previously described [70]. Immediate post mortem left-ventricular cardiac samples were fixed in formalin (3.5–3.7%) for 6 days, dehydrated, and embedded in paraffin blocks. Paraffin sections (3–5 μm) were cut, de-paraffinized in xylene, and rehydrated in a graded series of ethanol (100% Ethanol (1 min), 100% Ethanol (5 min), 90% Ethanol (3 min), 70% Ethanol (5 min)) and deionized water. Heat-induced antigen retrieval was performed by heating up the slides in a microwave for 2 times 3 min in 10 mM citrate solution (pH 6). After cooling back to room temperature, the slides were blocked for 20 min with 10% normal goat serum (Jackson ImmunoResearch Laboratories, Inc., West Grove, PA, UK) before incubating for 1 h with the following primary antibodies: β_2_-adrenergic receptor (ADRB2 rabbit polyclonal antibody, proteintech 13096-1-AP), mitochondrial sulfide quinone oxidoreductase (SQRDL rabbit polyclonal antibody, proteintech 17256-1-AP), NADH dehydrogenase (ubiquinone) 1 beta subcomplex 8 (NDUFB8 rabbit polyclonal antibody, proteintech 14794-1-AP), succinate dehydrogenase complex subunit A (SDHA rabbit polyclonal antibody 14865-1-AP), ubiquinol-cytochrome c reductase Rieske iron-sulfur polypeptide 1 (UQCRFS1 rabbit polyclonal antibody, proteintech 18443-1-AP), cytochrome c oxidase subunit Va (COX5A polyclonal antibody, proteintech 11448-1-AP), nitrotyrosine (Anti-Nitrotyrosine polyclonal antibody, Millipore, MA, USA, AB5411). Primary antibody detection was performed by a Dako REAL detection system (anti-mouse, anti-rabbit; alkaline phosphatase conjugated) and visualized with red chromogen (Dako REAL; Dako, Agilent Technologies, Santa Clara, CA, USA) followed by counterstaining with hematoxylin (Sigma). Washing steps (TBS 1 min, TBS Tween 3 min, TBS 1 min) were performed after primary antibody incubation (1 h), Dako REAL, Biotinylated Secondary Antibodies incubation (30 min), Dako REAL Streptavidin Alkaline Phosphatase (AP) incubation (30 min), and Dako REAL red chromogen. A Zeiss Axio Imager A1 microscope with a 10× objective was used for visualization of the slides. Two representative 800,000 μm^2^ sections per slide were graded for quantification of the red chromogen using the Zen Image Analysis Software 3.0 blue edition (Zeiss, Oberkochen, Germany). All primary antibodies were titrated to their optimal dilutions within the range recommended by the manufacturer. Antibody specificity had been confirmed in NCBI BLAST searches (courtesy of the U.S. National Library of Medicine, https://blast.ncbi.nlm.nih.gov/Blast.cgi, November 2023). We compared immunogen sequences of the used antibodies to the *Sus scrofa* database. Immunohistochemistry results are presented as % positive area.

### 4.4. Data Analysis

Since there was neither any effect of the group assignment to the “control” and “early life stress (ELS)” groups nor any sex-specific effect on plasma catecholamine concentrations, blood markers of ROS production or oxidative stress, nor on tissue mitochondrial respiration or protein expression, we performed a pooled analysis of all 12 individual animals. Nevertheless, individual animals are presented according to their original group assignment, i.e., male (closed squares)/female (open circles) and presence (red)/absence (blue symbols) of ELS. For the original experiment [27], a power analysis had not been feasible due to (i) the unavailability of appropriate literature data, and the fact that (ii) our approach of “mild” early weaning had allowed avoidance of any clinical symptoms, phenotype differences, and/or effects on growth, body weight, and behavior of the individual animals. Accordingly, the Animal Care Committee of the Universität Ulm and the Federal Authorities for Animal Research (Regierungspräsidium Tübingen) deemed our study as “exploratory” allowing for *n* = 6 per group only. For the complete set of data, normal distribution was tested by Shapiro-Wilk test, and consequently, all data are presented as median (interquartile range) unless stated differently. Correlation coefficients were calculated according to Pearson for linear modeling. Non-linear correlation coefficients were calculated according to Spearman; the model calculated used the following general formula: *y* = *x*_plateau_ + (*y_x_*_=0_ − *x*_plateau_) · *e^k^* ^· *x*^. All statistical analyses were carried out with Origin 2019b (9.6.5) (OriginLab Corporation, Northampton, MA, USA).

## 5. Conclusions

The present study aimed to answer the question whether variations in plasma catecholamine levels within the normal range may impact myocardial β-receptor expression and/or mitochondrial respiratory activity. The main findings of this study were that even physiological catecholamine concentrations showed significant inverse relationships with both myocardial mitochondrial respiratory activity and tissue expression of the β_2_-adrenergic receptor, whereas no relation was found between catecholamine concentrations and the expression of mitochondrial respiratory complexes. Hence our study suggests that even variations in the plasma catecholamine concentrations within this normal physiological range, e.g., resulting from psychological and/or physical stress, may affect the cardiac tissue β_2_-adrenergic receptor expression and thereby possibly the response of cardiac tissue energy metabolism to β-adrenoceptor agonists.

## Figures and Tables

**Table 1:** Plasma adrenaline, noradrenaline, isoprostane, whole-blood superoxide anion (O_2_^•−^), and DNA strand breaks (“tail moment” in the comet assay). All data are median (interquartile range), *n* = 12. Note that in one animal, the adrenaline concentration was below the detection limit; for this animal the threshold value of 15 pg/mL was recorded.

Adrenaline [pg/mL]	Noradrenaline [pg/mL]	Isoprostane [pg/mL]	O_2_^•−^ [μmol/L]	Tail Moment
75 (28; 143)	127 (92; 228)	43 (39; 47)	3.8 (3.5; 4.6)	0.16 (0.15; 0.18)

**Table 2:** Maximum respiratory capacity in the coupled (OxPhos) and the maximum electron transfer capacity in the uncoupled state (ETC) of the myocardial mitochondrial respiratory activity, presented as JO_2_, and tissue protein expression of the mitochondrial NADH dehydrogenase (ubiquinone oxidoreductase, complex I) and succinate dehydrogenase (complex II) and the β_2_-adrenergic receptor, presented as % positive area staining. All data are median (interquartile range), *n* = 12.

JO_2_ -OxPhos [pmol/s/mg_tissue_]	JO_2_-ETC [pmol/s/mg_tissue_]	Complex I [%]	Complex II [%]	β_2_-Adrenoreceptor [%]
194 (110; 223)	222 (158; 261)	86 (78; 89)	71 (60; 82)	83 (70; 89)

## Data Availability

The data presented in this study are available on request from the corresponding author.

## References

[1] Witkowska M., Halawa B. (1989). Beta-adrenergic receptors and catecholamines in acute myocardial infarction. Mater. Med. Pol..

[2] Merlet P., Delforge J., Syrota A., Angevin E., Mazière B., Crouzel C., Valette H., Loisance D., Castaigne A., Randé J.L. (1993). Positron Emission Tomography with ^11^C CGP-12177 to Assess β-Adrenergic Receptor Concentration in Idiopathic Dilated Cardiomyopathy. Circulation.

[3] Scharf S.M., Chen L., Slamowitz D., Rao P.S. (1996). Effects of Continuous Positive Airway Pressure on Cardiac Output and Plasma Norepinephrine in Sedated Pigs. J. Crit. Care.

[4] Kribbs S.B., Merritt W.M., Clair M.J., Krombach R.S., Houck W.V., Dodd M.G., Mukherjee R., Spinale F.G. (1998). Amlodipine Monotherapy, Angiotensin-Converting Enzyme Inhibition, and Combination Therapy with Pacing-Induced Heart Failure. Hypertension.

[5] Spinale F.G., de Gasparo M., Whitebread S., Hebbar L., Clair M.J., Melton D.M., Krombach R.S., Mukherjee R., Iannini J.P., O S.J. (1997). Modulation of the Renin-Angiotensin Pathway through Enzyme Inhibition and Specific Receptor Blockade in Pacing-Induced Heart Failure: I. Effects on Left Ventricular Performance and Neurohormonal Systems. Circulation.

[6] King M.K., Coker M.L., Goldberg A., McElmurray J.H., Gunasinghe H.R., Mukherjee R., Zile M.R., O’Neill T.P., Spinale F.G. (2003). Selective Matrix Metalloproteinase Inhibition with Developing Heart Failure: Effects on Left Ventricular Function and Structure. Circ. Res..

[7] New R.B., Sampson A.C., King M.K., Hendrick J.W., Clair M.J., McElmurray J.H., Mandel J., Mukherjee R., de Gasparo M., Spinale F.G. (2000). Effects of Combined Angiotensin II and Endothelin Receptor Blockade with Developing Heart Failure: Effects on Left Ventricular Performance. Circulation.

[8] Brown D.A., Perry J.B., Allen M.E., Sabbah H.N., Stauffer B.L., Shaikh S.R., Cleland J.G.F., Colucci W.S., Butler J., Voors A.A. (2017). Expert consensus document: Mitochondrial function as a therapeutic target in heart failure. Nat. Rev. Cardiol..

[9] Liaudet L., Calderari B., Pacher P. (2014). Pathophysiological mechanisms of catecholamine and cocaine-mediated cardiotoxicity. Heart Fail. Rev..

[10] Sorescu D., Griendling K.K. (2002). Reactive Oxygen Species, Mitochondria, and NAD(P)H Oxidases in the Development and Progression of Heart Failure. Congest. Heart Fail..

[11] Lemieux H., Semsroth S., Antretter H., Höfer D., Gnaiger E. (2011). Mitochondrial respiratory control and early defects of oxidative phosphorylation in the failing human heart. Int. J. Biochem. Cell Biol..

[12] Sabbah H.N., Sharov V., Riddle J.M., Kono T., Lesch M., Goldstein S. (1992). Mitochondrial Abnormalities in Myocardium of Dogs with Chronic Heart Failure. J. Mol. Cell. Cardiol..

[13] Lefroy D.C., de Silva R., Choudhury L., Uren N.G., Crake T., Rhodes C.G., Lammertsma A.A., Boyd H., Patsalos P.N., Nihoyannopoulos P. (1993). Diffuse Reduction of Myocardial Beta-Adrenoceptors in Hypertrophic Cardiomyopathy: A Study with Positron Emission Tomography. J. Am. Coll. Cardiol..

[14] Triposkiadis F., Karayannis G., Giamouzis G., Skoularigis J., Louridas G., Butler J. (2009). The Sympathetic Nervous System in Heart Failure: Physiology, Pathophysiology, and Clinical Implications. J. Am. Coll. Cardiol..

[15] Perez D.M. (2021). Targeting Adrenergic Receptors in Metabolic Therapies for Heart Failure. Int. J. Mol. Sci..

[16] Bristow M.R., Ginsburg R., Umans V., Fowler M., Minobe W., Rasmussen R., Zera P., Menlove R., Shah P., Jamieson S. (1986). β_1_- and β_2_-Adrenergic-Receptor Subpopulations in Nonfailing and Failing Human Ventricular Myocardium: Coupling of Both Receptor Subtypes to Muscle Contraction and Selective β_1_-Receptor Down-Regulation in Heart Failure. Circ. Res..

[17] Brodde O.E. (1993). Beta-arenoceptors in cardiac disease. Pharmacol. Ther..

[18] Gengo P.J., Bowling N., Wyss V.L., Hayes J.S. (1988). Effects of Prolonged Phenylephrine Infusion on Cardiac Adrenoceptors and Calcium Channels. J. Pharmacol. Exp. Ther..

[19] Neri M., Cerretani D., Fiaschi A.I., Laghi P.F., Lazzerini P.E., Maffione A.B., Micheli L., Bruni G., Nencini C., Giorgi G. (2007). Correlation between cardiac oxidative stress and myocardial pathology due to acute and chronic norepinephrine administration in rats. J. Cell. Mol. Med..

[20] Izem-Meziane M., Djerdjouri B., Rimbaud S., Caffin F., Fortin D., Garnier A., Veksler V., Joubert F., Ventura-Clapier R. (2012). Catecholamine-induced cardiac mitochondrial dysfunction and mPTP opening: Protective effect of curcumin. Am. J. Physiol. Heart Circ. Physiol..

[21] Sone T., Miyazaki Y., Ogawa K., Satake T. (1984). Effects of Excessive Noradrenaline on Cardiac Mitochondrial Calcium Transport and Oxidative Phosphorylation. Jpn. Circ. J..

[22] Todd G.L., Baroldi G., Pieper G.M., Clayton F.C., Eliot R.S. (1985). Experimental catecholamine-induced myocardial necrosis. II. Temporal development of isoproterenol-induced contraction band lesions correlated with ECG, hemodynamic and biochemical changes. J. Mol. Cell. Cardiol..

[23] Boillot A., Massol J., Maupoil V., Grelier R., Capellier G., Berthelot A., Barale F. (1996). Alterations of myocardial and vascular adrenergic receptor-mediated responses in *Escherichia coli*-induced septic shock in the rat. Crit. Care Med..

[24] Mizumachi K., Yahagi M., Kawabata H., Tezuka S., Honda T., Okada K. (1991). Decreased Beta-Adrenergic Receptor Density in Rat Myocardium during Hemorrhagic Shock. J. Anesth..

[25] Kolseth S.M., Wahba A., Kirkeby-Garstad I., Aro S., Nordgaard H., Høydal M., Rognmo Ø., Nordhaug D. (2012). A dose-response study of levosimendan in a porcine model of acute ischaemic heart failure. Eur. J. Cardiothorac. Surg..

[26] Rosca M.G., Vazquez E.J., Kerner J., Parland W., Chandler M.P., Stanley W., Sabbah H.N., Hoppel C.L. (2008). Cardiac mitochondria in heart failure: Decrease in respirasomes and oxidative phosphorylation. Cardiovasc. Res..

[27] Münz F., Wolfschmitt E.-M., Zink F., Abele N., Hogg M., Hoffmann A., Gröger M., Calzia E., Waller C., Radermacher P. (2023). Porcine blood cell and brain tissue energy metabolism: Effects of “early life stress”. Front. Mol. Biosci..

[28] Kim Y.-H., Kim K.-Y. (2021). Effect of air cleaner on stress hormones of pig and pork quality. J. Anim. Sci. Technol..

[29] Basu S. (2008). F_2_-Isoprostanes in Human Health and Diseases: From Molecular Mechanisms to Clinical Implications. Antioxid. Redox Signal..

[30] Gajski G., Langie S., Zhanataev A. (2020). Recent applications of the Comet Assay: A report from the International Comet Assay Workshop 2019. Toxicol. Lett..

[31] Milić M., Ceppi M., Bruzzone M., Azqueta A., Brunborg G., Godschalk R., Koppen G., Langie S., Møller P., Teixeira J.P. (2021). The hCOMET project: International database comparison of results with the comet assay in human biomonitoring. *Baseline frequency of DNA damage and effect of main confounders*. Mutat. Res. Rev. Mutat. Res..

[32] Ewelina G., Krzysztof S., Marek M., Krzysztof K. (2017). Blood free Radicals Concentration Determined by Electron Paramagnetic Resonance Spectroscopy and Delayed Cerebral Ischemia Occurrence in Patients with Aneurysmal Subarachnoid Hemorrhage. Cell Biochem. Biophys..

[33] Mannaerts D., Faes E., Gielis J., van Craenenbroeck E., Cos P., Spaanderman M., Gyselaers W., Cornette J., Jacquemyn Y. (2018). Oxidative stress and endothelial function in normal pregnancy versus pre-eclampsia, a combined longitudinal and case control study. BMC Pregnancy Childbirth.

[34] Heinonen I., Sorop O., van Dalen B.M., Wüst R.C.I., van de Wouw J., de Beer V.J., Octavia Y., van Duin R.W.B., Hoogstrate Y., Blonden L. (2020). Cellular, mitochondrial and molecular alterations associate with early left ventricular diastolic dysfunction in a porcine model of diabetic metabolic derangement. Sci. Rep..

[35] Jarkovska D., Markova M., Horak J., Nalos L., Benes J., Al-Obeidallah M., Tuma Z., Sviglerova J., Kuncova J., Matejovic M. (2018). Cellular Mechanisms of Myocardial Depression in Porcine Septic Shock. Front. Physiol..

[36] Strømholm T., Aadahl P., Saether O., Myking O., Myhre H. (1999). Excessive Increase in Circulating Catecholamines during Cross-clamping of the Descending Thoracic Aorta in Pigs. Int. J. Angiol..

[37] Dalin A.M., Magnusson U., Häggendal J., Nyberg L. (1993). The Effect of Transport Stress on Plasma Levels of Catecholamines, Cortisol, Corticosteroid-Binding Globulin, Blood Cell Count, and Lymphocyte Proliferation in Pigs. Acta Vet. Scand..

[38] Zhang N., Feng B., Ma X., Sun K., Xu G., Zhou Y. (2019). Dapagliflozin improves left ventricular remodeling and aorta sympathetic tone in a pig model of heart failure with preserved ejection fraction. Cardiovasc. Diabetol..

[39] Spinale F.G., Mukherjee R., Krombach R.S., Clair M.J., Hendrick J.W., Houck W.V., Hebbar L., Kribbs S.B., Zellner J.L., Dodd M.G. (1998). Chronic Amlodipine Treatment during the Development of Heart Failure. Circulation.

[40] Klemcke H.G., Nienaber J.A., Hahn G.L. (1989). Plasma Adrenocorticotropic Hormone and Cortisol in Pigs: Effects of Time of Day on Basal and Stressor-Altered Concentrations. Proc. Soc. Exp. Biol. Med..

[41] Waller C., Rhee D.-S., Gröger M., Rappel M., Maier T., Müller M., Rottler E., Nerz K., Nerz C., Brill S. (2020). Social Stress-Induced Oxidative DNA Damage Is Related to Prospective Cardiovascular Risk. J. Clin. Med..

[42] Barnes P.J., Ind P.W., Brown M.J. (1982). Plasma Histamine and Catecholamines in Stable Asthmatic Subjects. Clin. Sci..

[43] Åkerstedt T., Gillberg M., Hjemdahl P., Sigurdson K., Gustavsson I., Daleskog M., Pollare T. (1983). Comparison of urinary and plasma catecholamine responses to mental stress. Acta Physiol. Scand..

[44] Kawabe H., Saito I., Hasegawa C., Nagano S., Saruta T. (1994). Circulatory and Plasma Catecholamine Responses to Mental Stress in Young Subjects with Two Different Types of Hypertension. Angiology.

[45] Boeck C., Koenig A.M., Schury K., Geiger M.L., Karabatsiakis A., Wilker S., Waller C., Gündel H., Fegert J.M., Calzia E. (2016). Inflammation in adult women with a history of child maltreatment: The involvement of mitochondrial alterations and oxidative stress. Mitochondrion.

[46] Boeck C., Krause S., Karabatsiakis A., Schury K., Gündel H., Waller C., Kolassa I.-T. (2018). History of child maltreatment and telomere length in immune cell subsets: Associations with stress- and attachment-related hormones. Dev. Psychopathol..

[47] Gumpp A.M., Behnke A., Ramo-Fernández L., Radermacher P., Gündel H., Ziegenhain U., Karabatsiakis A., Kolassa I.-T. (2023). Investigating mitochondrial bioenergetics in peripheral blood mononuclear cells of women with childhood maltreatment from post-parturition period to one-year follow-up. Psychol. Med..

[48] Karrasch S., Matits L., Bongartz W., Mavioğlu R.N., Gumpp A.M., Mack M., Tumani V., Behnke A., Steinacker J.M., Kolassa I.-T. (2023). An exploratory study of hypnosis-induced blood count changes in chronically stressed individuals. Biol. Psychol..

[49] Karrasch S., Mavioğlu R.N., Matits L., Gumpp A.M., Mack M., Behnke A., Tumani V., Karabatsiakis A., Bongartz W., Kolassa I.-T. (2023). Randomized controlled trial investigating potential effects of relaxation on mitochondrial function in immune cells: A pilot experiment. Biol. Psychol..

[50] Berthon D., Herpin P., Bertin R., de Marco F., le Dividich J. (1996). Metabolic Changes Associated with Sustained 48-Hr Shivering Thermogenesis in the Newborn Pig. Comp. Biochem. Physiol. B Biochem. Mol. Biol..

[51] Hiemstra J.A., Gutiérrez-Aguilar M., Marshall K.D., McCommis K.S., Zgoda P.J., Cruz-Rivera N., Jenkins N.T., Krenz M., Domeier T.L., Baines C.P. (2014). A new twist on an old idea part 2: Cyclosporine preserves normal mitochondrial but not cardiomyocyte function in mini-swine with compensated heart failure. Physiol. Rep..

[52] Communal C., Ribuot C., Durand A., Demenge P. (1998). Myocardial β-adrenergic reactivity in volume overload-induced cardiac hypertrophy in the rat. Fundam. Clin. Pharmacol..

[53] Forster C., Naik G.O., Larosa G. (1994). Myocardial β-adrenoceptors in pacing-induced heart failure: Regulation by enalapril?. Can. J. Physiol. Pharmacol..

[54] Melis M.J., Miller M., Peters V.B.M., Singer M. (2023). The role of hormones in sepsis: An integrated overview with a focus on mitochondrial and immune cell dysfunction. Clin. Sci. (Lond).

[55] Kruk J., Kotarska K., Aboul-Enein B.H. (2020). Physical Exercise and Catecholamines Response: Benefits and Health Risk—Possible Mechanisms. Free Radic. Res..

[56] Rump A.F.E., Schierholz J., Klaus W. (2002). Studies on the Cardiotoxicity of Noradrenaline in Isolated Rabbit Hearts. Arzneimittelforschung.

[57] Rump A.F., Klaus W. (1994). Evidence for norepinephrine cardiotoxicity mediated by superoxide anion radicals in isolated rabbit hearts. Naunyn Schmiedeberg’s Arch. Pharmacol..

[58] van ‘t Erve T.J., Kadiiska M.B., London S.J., Mason R.P. (2017). Classifying oxidative stress by F_2_-isoprostane levels across human diseases: A meta-analysis. Redox Biol..

[59] Moeser A.J., Pohl C.S., Rajput M. (2017). Weaning stress and gastrointestinal barrier development: Implications for lifelong gut health in pigs. Anim. Nutr..

[60] McLamb B.L., Gibson A.J., Overman E.L., Stahl C., Moeser A.J. (2013). Early Weaning Stress in Pigs Impairs Innate Mucosal Immune Responses to Enterotoxigenic *E. coli* Challenge and Exacerbates Intestinal Injury and Clinical Disease. PLoS ONE.

[61] Moeser A.J., Ryan K.A., Nighot P.K., Blikslager A.T. (2007). Gastrointestinal dysfunction induced by early weaning is attenuated by delayed weaning and mast cell blockade in pigs. Am. J. Physiol. Gastrointest. Liver Physiol..

[62] Smith F., Clark J.E., Overman B.L., Tozel C.C., Huang J.H., Rivier J.E.F., Blikslager A.T., Moeser A.J. (2010). Early weaning stress impairs development of mucosal barrier function in the porcine intestine. Am. J. Physiol. Gastrointest. Liver Physiol..

[63] Zink F., Vogt J., Wachter U., Hartert J., Horchler M., Zhang X., Hezel F., Kapapa T., Datzmann T., Hoffmann A. (2021). Effects of acute subdural hematoma-induced brain injury on energy metabolism in peripheral blood mononuclear cells. Shock.

[64] Ost M., Doerrier C., Gama-Perez P., Moreno-Gomez S. (2018). Analysis of mitochondrial respiratory function in tissue biopsies and blood cells. Curr. Opin. Clin. Nutr. Metab. Care.

[65] Speit G., Hartmann A. (2006). The comet assay: A Sensitive Genotoxicity Test for the Detection of DNA Damage and Repair. Methods Mol. Biol..

[66] Hinson J.A., Michael S.L., Ault S.G., Pumford N.R. (2000). Western Blot Analysis for Nitrotyrosine Protein Adducts in Livers of Saline-Treated and Acetaminophen-Treated Mice. Toxicol. Sci..

[67] Datzmann T., Kapapa T., Scheuerle A., McCook O., Merz T., Unmuth S., Hoffmann A., Mathieu R., Mayer S., Mauer U.M. (2019). In-depth characterization of a long-term, resuscitated model of acute subdural hematoma-induced brain injury. J. Neurosurg..

[68] Hartmann C., Loconte M., Antonucci E., Holzhauser M., Hölle T., Katzsch D., Merz T., McCook O., Wachter U., Vogt J.A. (2017). Effects of Hyperoxia during Resuscitation from Hemorrhagic Shock in Swine with Preexisting Coronary Artery Disease. Crit. Care Med..

[69] Merz T., Lukaschewski B., Wigger D., Rupprecht A., Wepler M., Gröger M., Hartmann C., Whiteman M., Szabo C., Wang R. (2018). Interaction of the hydrogen sulfide system with the oxytocin system in the injured mouse heart. Intensive Care Med. Exp..

[70] Merz T., Denoix N., Wigger D., Waller C., Wepler M., Vettorazzi S., Tuckermann J., Radermacher P., McCook O. (2020). The Role of Glucocorticoid Receptor and Oxytocin Receptor in the Septic Heart in a Clinically Relevant, Resuscitated Porcine Model with Underlying Atherosclerosis. Front. Endocrinol. (Lausanne).

